# Associations between genetic loci, environment factors and mental disorders: a genome-wide survival analysis using the UK Biobank data

**DOI:** 10.1038/s41398-022-01782-8

**Published:** 2022-01-11

**Authors:** Peilin Meng, Jing Ye, Xiaomeng Chu, Bolun Cheng, Shiqiang Cheng, Li Liu, Xuena Yang, Chujun Liang, Feng Zhang

**Affiliations:** Key Laboratory of Trace Elements and Endemic Diseases of National Health and Family Planning Commission, School of Public Health, Health Science Center, Xi’an Jiaotong University, Xi’an, China

**Keywords:** Genomics, Molecular neuroscience

## Abstract

It is well-accepted that both environment and genetic factors contribute to the development of mental disorders (MD). However, few genetic studies used time-to-event data analysis to identify the susceptibility genetic variants associated with MD and explore the role of environment factors in these associations. In order to detect novel genetic loci associated with MD based on the time-to-event data and identify the role of environmental factors in them, this study recruited 376,806 participants from the UK Biobank cohort. The MD outcomes (including overall MD status, anxiety, depression and substance use disorders (SUD)) were defined based on in-patient hospital, self-reported and death registry data collected in the UK Biobank. SPACOX approach was used to identify the susceptibility loci for MD using the time-to-event data of the UK Biobank cohort. And then we estimated the associations between identified candidate loci, fourteen environment factors and MD through a phenome-wide association study and mediation analysis. SPACOX identified multiple candidate loci for overall MD status, depression and SUD, such as rs139813674 (*P* value = 8.39 × 10^–9^, ZNF684) for overall MD status, rs7231178 (DCC*, P* value = 2.11 × 10^–9^) for depression, and rs10228494 (FOXP2, *P* value = 6.58 × 10^–10^) for SUD. Multiple environment factors could influence the associations between identified loci and MD, such as confide in others and felt hated. Our study identified novel candidate loci for MD, highlighting the strength of time-to-event data based genetic association studies. We also observed that multiple environment factors could influence the association between susceptibility loci and MD.

## Introduction

Mental health problems have been the leading cause of disability globally, from 1990 to 2010, the burden of mental and substance use disorders (SUD) increased by 37.6% [1]. About 2.64 million and 2.84 million people worldwide are estimated to suffer from depression and anxiety [2], which have attracted widespread international attention. However, even growing attention has been taken to global mental health, the burden of disease attributable to mental disorders(MD) continues to increase [3]. Furthermore, it is estimated that more than 70% of persons worldwide who need mental health services lack access to care [4]. Given the limited public expenditure on mental health by national governments in low- and middle-income countries [5] and the inefficient treatment of MD, efforts to explore the identification and prevention of MD are critical.

It is well accepted that genetic factor playes an indispensable role in the development of MD. For instance, twin and family studies estimated that approximately 31–42% of unipolar depression [6] and 30–60% of anxiety disorder [7] could be explained by genetic factors. Hydel et al. conducted a recent meta-analysis of depression genome-wide association study (GWAS) on 807,553 individuals (246,363 cases and 561,190 controls) from the three largest genome-wide association studies of depression [8], and identified 102 independent variants. However, traditional GWAS approach generally focused on MD susceptibility utilizing cross-sectional data rather than MD survival utilizing time-to-event data.

Genome-wide survival analysis estimates the genetic effects on the disease risk and the age of onset diseases. Tang et al. conducted a genome-wide survival analysis using overall survival data from MD Anderson Cancer Center and discovered three SNPs with genome-wide significant [9]. Huang et al. using Cox model screened and validated multiple significant SNPs associated with overall survival of non-small-cell lung cancer [10]. However, to our knowledge, few studies investigated the association of SNPs with MD survival using genome-wide survival analysis, partly because of the unavailability of time-to-event data and computational cost. Time-stamped longitudinal data of MD from UK Biobank enable us to explore genes influencing age at onset utilizing the new SPACOX approach, a faster and more accurate approach compared to the standard approach of Cox proportional hazards (PH) regression model. SPACOX is suitable for a genome-wide scale single-variant time-to-event data analysis while retaining well-controlled type I error rates and powers, which fits a null Cox PH model only once for genome-wide analysis, greatly improving the computational efficiency [11]. Due to the scalability to analyze hundreds of thousands of samples and calibration for common, low-frequency, and rare variants, SPACOX will facilitate the genome-wide time-to-event data analysis in large biobanks and contribute to the discovery of the genetic causes underlying complex diseases [11].

It is universally acknowledged that increased knowledge of the interaction of genetic and environmental risk factors is vital for a more comprehensive understanding the etiology of mental disorders. However, typically, geneticists pay little attention in the study of environmental risk factors, often contribute environmental influences to random error, while behavioral scientists are interested in environmental risk factors and put rare emphasis on issues either related to genes or to interactions [12]. It is therefore important to grasp how and what extent environmental factors affect mental health outcomes. A comprehensive twin meta-analysis indicated that environmental effects account for a 55% to 66% risk for major depression, 32% risk for bipolar disorder, and 23% risk for schizophrenia [13]. Another meta-analytical reviews have also suggested that psychotic syndromes is associated with early life adversity, growing up in an urban environment, and minority group position [14]. Besides, a network approach to environmental impact in psychotic disorder indicated the existence of specific paths between environmental factors and symptoms [15]. MD is phenotypically correlated with many environment factors, such as sleep behaviors [16], stress [17], childhood traumatic events [18] and social support [19]. There is a growing amount of psychological and physiological evidence that sleep and mental health are closely associated, and indeed may have reciprocal and mutually facilitating effects [20]. Stress is defined as the feeling of being overwhelmed or unable to cope with mental or emotional pressure, which has a causal link with mental illness. It’s reported that stressful life events are cause of the onset of depression [21]. What’s more, childhood trauma events can also trigger strong physical and emotional responses, which may persist long after the event passes and affect their daily lives and emotional well-being for years or even decades after the triggering event. Childhood trauma are related to anxiety and depressive disorders [22] and seem to account for one half to two third of serious problems with drug use [23]. As for social support, which can play an important role in creating, maintaining, and promoting health, is an important factor that can affect mental health [24]. About 25% of employed individuals engage in shift work, that is associated with considerable impacts on sleep, depressed mood and anxiety, substance use, impairments in cognition, lower quality of life, and even suicidal ideation [25]. Among various environmental factors, poverty related to income also has been widely concerned. Socioeconomic deprivation has been defined as a “state of observable and demonstrable disadvantage relative to the local community or the wider society to which an individual, family or group belongs.” by Townsend et al. [26], suggesting its advantage in measuring health inequality. Townsend deprivation index (TDI) is a popular measure of material deprivation based on four key variables: unemployment, non-home ownership, non-car ownership, and household overcrowding, which has been validated and applied in population studies in the United Kingdom, and higher scores represents higher levels of poverty [26]. TDI has been often used as covariates, such as in the longitudinal study investigating the impact of sleep duration and fragmentation on physical and mental health [27]. In our previous study evaluating the epidemiologic associations between the TDI and common psychiatric disorders or traits, we found that the trends of association remained still significant after adjusting for household income, suggesting the distinct role of TDI in relation to psychiatric diseases independent of income poverty [28]. Interestingly, Culver et al. pointed out that environmental exposures could modify the association between causal genetic variants and human disease by providing the necessary substrates or exerting an influence on the effect of specific genes [29]. It is necessary to investigate the effect of environmental risks affecting the associations between causal genetic variants and MD, to help to identify shared risk factors, mechanisms and consequences of MD [30].

In this study, we utilized the large time-to-events onset MD data to identify candidate loci for MD (included overall MD status, depression, anxiety and SUD) through SPACOX, respectively. Considering the influence of environment factors, a PheWAS and mediation analysis were then performed to estimate the effect of these factors modifying association between causal genetic variants and MD.

## Methods and materials

### UK Biobank cohort

Between 2006 and 2010, over 500,000 individuals aged 40–69 years were enrolled into the UK Biobank, a large and population-based prospective cohort study (http://www.ukbiobank.ac.uk/about-biobank-uk/) [31]. The participants were assessed in 22 assessment centers throughout the UK, covering a broad distribution across England, Wales and Scotland [31]. The broadly distributed exposures of the UK Biobank ensured the generalizable associations between baseline characteristics and health outcomes. The participants undergone a variety of physical and functional measurements, as described elsewhere [31]. The UK Biobank study was approved by the North West Multi-Centre Research Ethics Committee and the participants signed consent for the use of their anonymous data and samples for any health-related studies [31]. This study was conducted with the permission (UKB application 46478) from the UK Biobank.

### Outcomes and follow-up

The MD outcomes were defined based on in-patient hospital, self-reported and death registry data collected in the UK Biobank. ICD-10 (International Classification of Diseases 10th revision) is a classification code for diseases, external causes of injury, signs and symptoms, and abnormal findings, which is applied for monitoring population health, assessing quality and standards of care, and receiving reimbursements for services [32]. The alphanumeric diagnosis codes represent for the diagnoses, symptoms, and signs. Self-reported measures of MD outcomes were obtained from responses to the verbal interview conducted at the initial assessment centre, which were recorded in the non-cancer illness item (variable 20002). We used ICD-10 codes F00-99 and self-reported codes 1286-1291, 1408-1410, 1469-1470, 1614-1616 as overall MD status, participants with any reported MD codes were recognized as overall MD status in the study. Depression, anxiety and substance use disorders (SUD) were also taken into investigation. Depression was defined as ICD-10 codes F32-33 and self-reported codes 1286. Anxiety was defined as ICD-10 codes F40-41 and self-reported codes 1287. SUD was defined as ICD-10 codes F10-19 and self-reported codes 1408-1410. And Participants with no MD were coded as 0. For participants who had the time of self-reported and hospital diagnosis, we defined the earlier time as the diagnosis time. Following-up time was calculated from the birth date to diagnosis of MD outcomes, death, or the end of follow-up on 18 February 2016, whichever occurred first. And the detailed definitions of outcomes are provided in the supplementary table S1.

### UK Biobank genotyping, imputation and quality control

In the UK Biobank, either the UK BiLEVE Array or the UK Biobank Axiom Array was used to genotype and these genotypes were subsequently imputed to the Haplotype Reference Consortium (HRC) reference panel [33] (version 1.1) and UK10K and 1000 Genomes project reference panels [34]. Details of the array design, genotyping and quality control procedures have been described elsewhere [34]. We removed the participants whose self-reported sex was inconsistent with genetic sex, or who were genotyped but not imputed, or who withdrew their consent and removed genetically related participants [35]. The individuals were restricted to only “White British”. After filters, 376,806 participants were included in this study.

### SPACOX analyses

SPACOX is a fast and accurate approach to perform genome-wide survival analysis, using a saddlepoint approximation implementation based on Cox PH regression model [11]. SPACOX could identified more loci for human diseases, compared to traditional GWAS based on a logistic regression framework [11]. R package “SPACox” was used to perform genome-wide SNP association testing [11], with age, sex and 10 principal components of population structure as covariates. We used a significance threshold as *P* value <1.25 × 10^−8^, based on the Bonferroni correction (5.00 × 10^–8^/4 = 1.25 × 10^–8^). Additional quality control filters were used to select high-quality SNPs: the SNPs with low call rates (<0.90), low Hardy–Weinberg equilibrium exact test *P* values (<0.001), or low minor allele frequencies (MAFs <0.01) was excluded. The Circular Manhattan plots of all results was performed using “CMplot” R script (https://github.com/YinLiLin/R-CMplot).

### Sensitivity analysis

Cox PH model was then used to verify the significant genetic variants from SPACOX through R package “Survival” with age, sex and10 principal components as covariates. And individual SNPs were recoded as 0, 1, and 2 [36] according to the number of risk alleles through the command “recodeA” of PLINK [37]. The significance threshold was set as *P* value <1.25 × 10^−8^.

### Environment factors definition

Based on the results of previous studies, we totally collected fourteen environmental risk factors for MD, which consist of five categories, including sleep behaviors [16], stress [17], childhood traumatic events [18], social support [19] and shift work [38]. Sleep behaviors consisted of short sleep, long sleep, morning chronotype, insomnia, snoring and daytime dozing, which were considered as the unhealthy sleep pattern and associated with bad health outcome [39]. Stress was defined based on the question of “In the last 2 years have you experienced any of the following?” in the screen questionnaire from the UK Biobank. Childhood traumatic events consisted of three variables, including felt hated, physically abused and sexually molested from participants’ self-report on a screen questionnaire. Social support was defined from three domains, including friends visit, confide in others and social activities. Shift work was defined based on the question of “Does your work involve shift work?”. The detailed definitions of environment factors were provided in the supplementary materials.

### Statistical models

It is well-accepted that both environment factors and genetic factor contribute to the development of MD [40]. We aimed to explore the roles of genetic susceptibility and environmental risk factors in the development of MD. Table 1 shows the used SNPs in the further analysis, which were significantly associated with MD. We used GLM function in R to test PheWAS associations between the SNPs and fourteen environment factors, based on the previous study [41]. In addition, for the observed significant associations between SNPs and environment factors, we conducted a causal mediation analysis to investigate whether these environment factors might have a mediate effect on these associations. For each model, we adjusted the covariates of age, sex, 10 principal components, smoke ever, alcohol ever and TDI. We used a significance threshold as *P* value <0.05. Detailed introduction of a causal mediation analysis and the selection of the SNPs in Table 1 were provided in the supplementary materials which includes Fig. S1.

**Table 1:** The associations between MD associated SNPs and MD through SPACOX and Cox PH model.

		N	SNP	CHR	*P*_SPACOX_	*P*_PH_	Associated diseases
Depression	DCC	53	rs7231178	18	2.11 × 10^−09^	2.23 × 10^−09^	Mirror movementsgaze palsy Familial horizontal, with progressive scoliosis familial horizontal, with progressive scoliosis
			rs8092490	18	2.74 × 10^−09^	2.81 × 10^−09^	
			rs146002209	18	3.74 × 10^−09^	3.62 × 10^−09^	
SUD	BIRC6	6	rs4019436	2	2.90 × 10^−09^	2.76 × 10^−09^	Brain cancer
			rs6543658	2	3.48 × 10^−09^	3.30 × 10^−09^	
			rs17428810	2	5.20 × 10^−09^	4.74 × 10^−09^	
	FOXP2	20	rs10228494	7	6.58 × 10^−10^	6.16 × 10^−10^	Childhood apraxia of speechspeech and communication disorders
			rs10262103	7	1.23 × 10^−09^	1.14 × 10^−09^	
			rs71149745	7	1.63 × 10^−09^	1.54 × 10^−09^	
	SEMA6D	6	rs281296	15	7.59 × 10^−09^	7.21 × 10^−09^	Kallmann syndrome
			rs281287	15	7.81 × 10^−09^	7.36 × 10^−09^	
			rs1025143	15	1.01 × 10^−08^	9.60 × 10^−09^	
	CELF4	2	rs1941955	18	8.85 × 10^−10^	8.56 × 10^−10^	Dysgraphia and speechcommunication di sorders
			rs11663050	18	5.43 × 10^−09^	5.26 × 10^−09^	
	XK	1	rs10228494	7	6.58 × 10^−10^	6.16 × 10^−10^	McLeod syndrome
MD	ZNF684	1	rs139813674	1	8.39 × 10^−09^	3.40 × 10^−09^	Herpes simplex virus 1 infection

*N* was the number of the SNPs that were mapped to the gene of MD. The information of the genes associated diseases from GeneCards (https://www.genecards.org/) [42].

*SUD* substance use disorders, *MD* mental disorders, *PH* Cox proportional hazards.

## Results

### Descriptive characteristics

376,806 participants were included in this study and 202,436 (53.7%) of them were women, mean age were 56.99 (standard deviation (SD) = 7.93) years old. During the follow-up, 56,863 MD cases (median 64.71 years) occurred in participants from the birth date, including 31,414 incident depression cases, 13,463 incident anxiety cases, 17,604 SUD cases.

### Associations between SNPs and MD

For depression, the SPACOX identified 57 variants with *P* value < 1.25 × 10^–8^(Fig. S2), and 53 of those were mapped to gene DCC with the most significant SNP of rs7231178 (*P* value = 2.11 × 10^–9^) (Fig. S2). For SUD, the SPACOX identified 76 variants with *P* value < 1.25 × 10^–8^(Fig. S2), which were mapped to five genes, such as BIRC6 (rs4019436, *P* value = 2.90 × 10^–9^), FOXP2 (rs10228494, *P* value = 6.58 × 10^–10^) and SEMA6D (rs281296, *P* value = 7.59 × 10^–9^). For anxiety, the SPACOX did not identify the variants with *P* value < 1.25 × 10^–8^(Fig. S3). For overall MD status, the SPACOX identified five variants with *P* value < 1.25 × 10^–8^(Fig. S4), such as rs139813674 at chromosome 1 (*P* value = 8.39 × 10^–9^, ZNF684).

Most of the significant SNPs from SPACOX were also significant with *P* value <1.25 × 10^–8^ through Cox PH model (Table S2-4 in the supplementary tables). Table 1 showed the detailed information based on GeneCards (https://www.genecards.org/) [42].

### Associations between the SNPs and environment factors

**Fig. 1:**
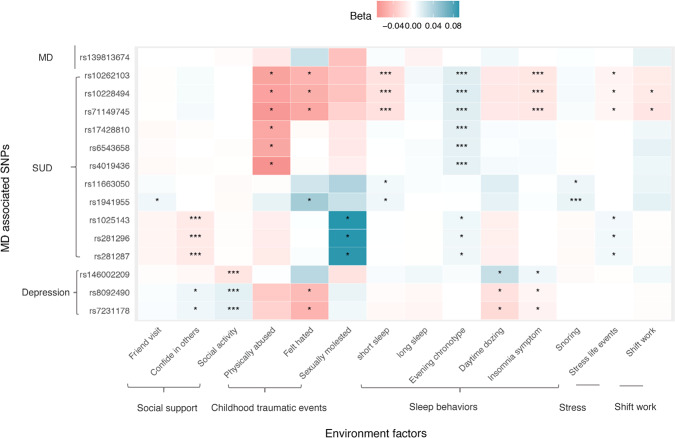
Heatmap for the associations between the SNPs and environment factors. *Additional details are provided in supplementary table S5. “*”represents *P* value <0.05, “***”represents *P* value <0.001.MD, mental disorders.

Figure 1 shows the results of the association between the SNPs and fourteen environment factors, additional details are provided in supplementary table S5. For depression, we observed that multiple SNPs were significantly associated with confide in others, social activity, felt hated, daytime dozing and insomnia symptom. For SUD, we observed that only long sleep was not significantly associated with the identified SNP of SUD. For the overall MD status, no SNP was significantly associated with the fourteen environment factors.

### Mediation analyses of environment factors

**Fig. 2:**
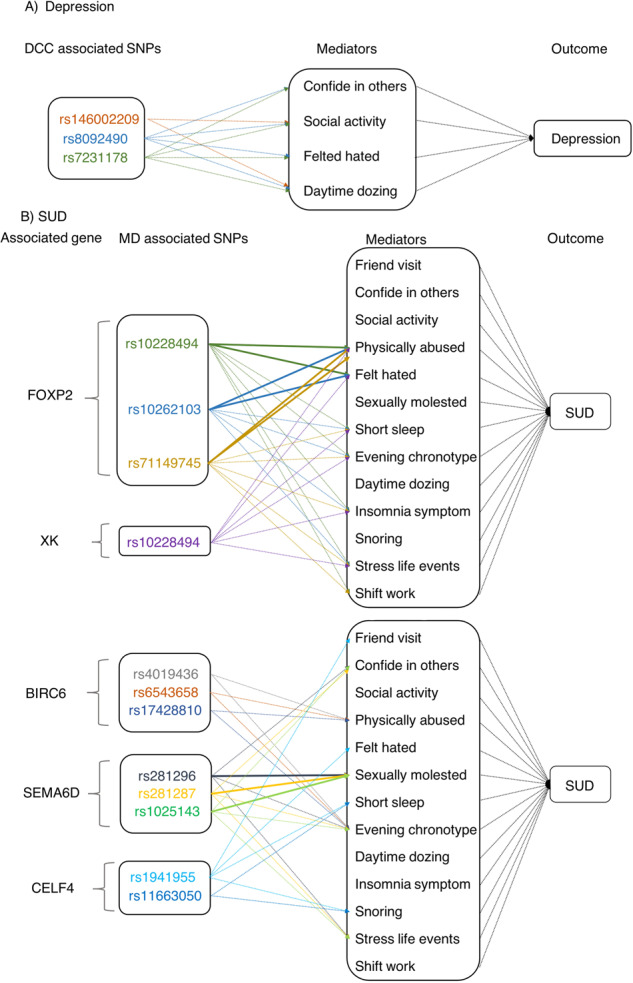
Mediation analyses results for environment factors. *Fig. 2 shows the environment factors play a mediating role between MD associated SNPs and MD diseases, including depression (**A**) and SUD (**B**). A virtual arrow represents a partial mediation and a solid arrow represents a full mediation. MD, mental disorders. MD, mental disorders; SUD, substance use disorders.

For the significant associations between the SNPs and environment factors, we conducted mediation analyses. We performed to explore the role of environment factors in these associations (Fig. 2). Firstly, we estimated the associations between the SNPs and MD through a logistic regression model, and supplementary table S6 shows that all results were significant. Secondly, we estimated the associations between environment factors and MD (depression and SUD) (Table S7 in supplementary tables), the results showed that depression was also associated with confide in others, social activity, felt hated and daytime dozing. SUD was also associated with friend visit, confide in others, social activity, felt hated, physically abused, sexually molested, short sleep, evening chronotype, insomnia symptom, stress life events and shift work. Combining with the previous significant results between SNPs and diseases, as well as SNPs and these factors, the results suggested that some of these factors had a mediating effect on the associations between significant SNPs and MD. In addition, we estimated the associations between SNPs and diseases with the factors as a covariate (Table S8 in supplementary tables), the results showed that physically abused and felt hated fully mediated the associations of SUD with rs71149745, rs10228494, and 10262103, and these three SNPs were mapped to FOXP2. And sexually molested fully mediated the associations of SUD with rs281287, rs281296 and rs1025143, which were mapped to SEMA6D.

## Discussion

In this study, a genome-wide survival analysis was conducted and we discovered some important candidate loci that were associated MD, such as DCC and FOXP2. Interestingly, these genes were associated with mental related diseases and nervous system function in the previous studies. For instance, DCC (DCC netrin 1 receptor), which encodes netrin 1 receptor, is considered as a cell-autonomous regulator for midline guidance in the central nervous system [43]. Li et al. investigated the association between autism spectrum disorder (ASD) and DCC of 231 ASD cases and 242 controls in Chinese Han, and indicated that haplotypes of seven SNPs in DCC may be implicated in ASD [44].

DCC also was reported to be associated with congenital mirror movements [45] and opioid addition [46], and animal experiment studies indicated that DCC could control corticospinal tract midline crossing in both human and mice [47]. Howard et al performed genome-wide significant gene-based hits in the meta-analysis of depression and reported that DCC was a significant gene consisting of 115 independent SNPs [48].

FOXP2 (forkhead box P2) encodes a member of the forkhead/winged-helix family of transcription factors, which expressed in fetal and adult brain [49]. And FOXP2 has been associated with language-related disorders, because the gene is required for proper development of speech and language regions of the brain during embryogenesis [49]. And Hickey et al. reported that FOXP2 chromatin alterations drive the maturation of excitatory cortical neurons and might be particularly important for the development of cortical circuits in neurodevelopmental disorders [50].

The study also found multiple genes which were indirectly associated neural development in the previous study, such as ZNF684. ZNF684(zinc finger protein 684), a protein coding gene, is related to the pathways of gene expression and herpes simplex virus 1 infection [42]. Interestingly, herpes simplex virus 1 has been proved to be associated with depression [51]. In view of these, we speculate that the genetic mechanism of depression and SUD might be related to the regulation of neural development. In addition, these genes were associated with age-related diseases, which might be the reason why they were identified by SPACOX. For instance, netrin-1-DCC signaling systems were associated with age-related macular degeneration [52] and FOXP2 was associated with age-dependent dormant resident progenitors [53].

Previous study suggested that environment factors have been suggested to be a mediator of causal genetic variant affecting the risk of human diseases [29]. Shen et al. conducted a phenome-wide association and suggested that depression polygenic risk score (PRS) was associated with many environmental variables, such as insomnia, nap, smoke and income [41]. In this study, after adjusting the effect of cigarette smoking, alcohol drinking and economic factor of Townsend deprivation index, we found that depression and SUD related SNPs were associated with multiple environment factors, including sleep behaviors, stress, childhood traumatic events and social support. At the same time, these environment factors were also associated with MD. The results suggested that depression and SUD related variants might identify a vulnerability to environmental risk exposures effects. Interestingly, further mediation analyses observed that physically abused, felt hated and sexually molested fully mediated the associations between SUD and identified significant SNPs, highlighting the impact of childhood traumatic events on MD. Warrier et al. also identified significant relationship between childhood traumatic and autism PRS, further, childhood traumatic event could moderate the effect of autism PRS on self-harm score [18]. And a previous review, including 65 publications, systematically collected all available research data and concluded that child abuse and family violence have a major impact on the anxiety, depression and SUD in adulthood [54]. Therefore, it is helpful to identify high risk group of trauma individuals and to reduce their occurrence.

In addition, mediation analyses results showed that physically abused and felt hated fully mediated the associations of SUD with rs71149745, rs10228494 and 10262103, and these three SNPs were mapped to FOXP2. And sexually molested fully mediated the associations of SUD with rs281287, rs281296, and rs1025143, which were mapped to SEMA6D. McCarthy et al. demonstrated that FOXP2 could influence the probability of people experience auditory verbal hallucinations in the presence and absence of childhood parental emotion abuse [55]. FOXP2 [50] and SEMA6D [56] are associated with the development of language, suggesting that they might influence the probability of people suffering SUD in the presence and absence of childhood trauma through regulating language development.

The key strength of the current study is that we conducted a genome-wide event time data analysis using SPACOX approach and identified some important candidate loci for depression, SUD and MD. Bi et al. indicated that SPACOX could identify the candidate loci that might be missed when event occurrence status during the follow-up period was defined as binary phenotype through a logistic regression framework [11]. Therefore, our results could provide more genetic information about MD. And we verified these candidate loci through PH and a logistic regression model, and the results supported our conclusion. Furthermore, we observed several behavioral and environmental factors that could mediate the effect genetic variants on MD, especially childhood trauma events. The results might help to find further mechanism of MD.

The study also has limitations. First of all, due to the limitation of middle-aged and white participants in the UK Biobank, researchers should take care when extend the conclusions to other populations with different ethnicities ad age distributions. Second, behavioral and environmental variables were collected by retrospective measurement based on self-reported online questionnaires, which might increase the likelihood of measurement error and recall bias. Further studies with multiple design strategies are eager to confirm our findings and clarify the potential mechanism underlying the associations observed in this study.

In summary, our genome-wide event time data analysis leads to a large increase in associated loci for depression, SUD and MD using the UK Biobank cohort. Further analyses indicate that environmental factors could moderate the effect of genetic variants on MD, especially childhood trauma. The study will advance understanding the genetic architect of MD and provide behavioral and environmental factors that might reducing the gene susceptibility of MD. Future studies are warranted to clarify the more detailed mechanism of identified loci and explore the physiology of how environment factors influence the association between susceptibility loci and MD.

## Supplementary information

Supplementary materials

Supplementary tables

## Data Availability

The UKB data are available through the UK Biobank Access Management System https://www.ukbiobank.ac.uk/. We will return the derived data fields following UKB policy; in due course, they will be available through the UK Biobank Access Management System.
