# Supplementary material for: Associations between genetic loci, environment factors and mental disorders: a genome-wide survival analysis using the UK Biobank data
Source: Transl Psychiatry. 2022 Jan 11;12:17. doi: 10.1038/s41398-022-01782-8 (PMC8752606; doi:10.1038/s41398-022-01782-8)
Supplement: Supplementary file 1 — Supplementary materials [file 41398_2022_1782_MOESM1_ESM.docx]

**Supplementary materials**

***Definitions of sleep behaviors***

Short sleep and long sleep were defined from the variable of sleep duration from the UK Biobank. Sleep duration was recorded as number of reported hours by asking ‘About how many hours sleep do you get in every 24 h? (include naps)’. And then we categorized the sleep duration as short sleep (<7 h/day), normal (7–8 h/day), and long (≥9 h/day) consistent with previous studies (1).

Chronotype preference was defined from the question “Do you consider yourself to be (1) definitely a “morning” person, (2) more a “morning” than “evening” person, (3) more an “evening” person than a “morning” person, (4) definitely an “evening” person. We recoded the participants who responded (1) and (2) as the code 0 and the last who responded (3) and (4) as code 1.

Insomnia symptom was assessed using the question “Do you have trouble falling asleep at night or do you wake up in the middle of the night”, the responders with the answer (1) never/rarely and (2) sometimes were recoded as code 0 and the responders with the answer (3) usually were recoded as code 1.

Snoring was obtained based on the question “Does your partner or a close relative or friend complain about your snoring?” with responses of (1) yes and (2) no. Snoring was recoded as code 1 and the last was recoded as code 0.

Daytime dozing was obtained by asking “How likely are you to doze off or fall asleep during the daytime when you don’t mean to? (e.g. when working, reading or diving)”, with the responses of (0) never/rarely, (1) sometimes, (2) often and (3) all the time. The participants who answered (0) and (1) were recoded as code 0 and the participants who answered (2) and (3) were recoded as code 1.

***Definitions of stress***

Stress was defined based on the question “In the last 2 years have you experienced any of the following?”. We recoded the participants as 1 with answer “yes” for (1) serious illness, injury or assault to yourself, (2) serious illness, injury or assault of a close relative, (3) death of a close relative, (4) death of a spouse or partner, (5) marital separation/divorce, (6) financial difficulties were used to reflect the stress life events as the previous introduced (2). And the participants with the answer (-7) none of the above were recoded as 0.

***Definitions of childhood traumatic events***

Felt hated (20487) was defined based on the question “When I was growing up…I felt that someone in my family hated me?”. Physically abused (20488) was defined based on the question “When I was growing up…people in my family hit me so hard that it left me with bruises or marks”. And Sexually molested (20490) was defined based on the question “When I was growing up…Someone molested me (sexually)”. Three variables with the responses of (0) never true, (1) rarely true, (2) sometimes true, (3) often, (4) very often true. We recoded the participants with answers (0), (1) and (2) as 0, and with answers (3) and (4) as 1, respectively.

***Definitions of social support***

Friend visit (1031) was defined based on the question "How often do you visit friends or family or have them visit you?". And we recoded the participants as 0, with answer “(4) about once a month, (5) once very few months, (6) never or almost never and (7) no friends outside household”, and recoded participants as 1, with answer “(1) almost daily, (2) 2-4 times a week and (3) about once a week”.

Confide in others (2110) was defined based on the question "How often are you able to confide in someone close to you?", with answers of (0) Never or almost never, (1) Once every few months, (2) About once a month, (3) About once a week, (4) 2-4 times a week and (5) Almost daily. And we recoded the participants as 0 with answer (1), (2) and (3), and recoded the participants as 1 with answer (4) and (5).

Social activity (6160) was defined based on the question "Which of the following do you attend once a week or more often? (You can select more than one)". We recoded the participants as 1 with answer “yes” for (1) Sports club or gym, (2) Pub or social club, (3) Religious group, (4) Adult education class, (5) Other group activity. And the participants with the answer (-7) None of the above were recoded as 0.

***Definitions of Shift work***

Shift work (826) was defined based on the question of "Does your work involve shift work?", with responses of (1) Never/ rarely, (2) Sometimes, (3) Usually, (4) Always. And we recoded the participants as 0, with answer (1) and (2), and recoded the participants as 1, with answer (3) and (4).

***Covariates***

Smoking and alcohol ever were defined as the amount of cigarette smoking and alcohol drinking of each individual reported, respectively. We coded participant status as 1 if a respondent reported that they were smoking current or previous and 0. Similarly, we coded alcohol drinking as 1 if they were smoking current or previous and 0. Townsend deprivation index (189) was calculated based on specific areas of output from previous national censuses. Each participant is assigned a score corresponding to the output area in which their postcode is located(3). It is a composite score based on four key variables: the percentage of unemployment, overcrowd households, households without a car and non-home ownership(3), which represent the deprivation index.

***Individual genotype conversion***

We used “recodeA” function of PLINK(4) to convert the genotype of individuals to 0,1,2, which using the allele frequency to transform the genotype, such as the heterozygous genotype is coded as 1, for the other genotypes, the more frequent genotype is coded as 0, and the less frequent genotype is coded as 2(5).

***The selection of the SNPs***

Table 1 showed the SNPs that we selected to use in the further analysis, based on the standard as follow: Firstly, we cataloged the significant SNPs and their corresponding genes for each disease with *P* value < 1.25 × 10^–8^. Secondly, for each gene, we selected the corresponding SNPs of the three first *P* value. If the number of SNPs were less than three, then less than three SNPs of this gene were used to perform the further analysis. For anxiety, it did not have the SNP with *P* value < 1.25 × 10^–8^, therefore, no further analysis for anxiety.

**A causal mediation analysis**

As the Figure S1 show the stepwise regression method, if W2 and W3 were both significant, then the mediating effect existed. If W1 is significant, it is partially mediated, otherwise it is fully mediated.

**Reference**

1. Fan M, Sun D, Zhou T, Heianza Y, Lv J, Li L, et al. (2020): Sleep patterns, genetic susceptibility, and incident cardiovascular disease: a prospective study of 385 292 UK biobank participants. *European Heart Journal*. 41:1182-1189.

2. Arnau-Soler A, Macdonald-Dunlop E, Adams MJ, Clarke T-K, MacIntyre DJ, Milburn K, et al. (2019): Genome-wide by environment interaction studies of depressive symptoms and psychosocial stress in UK Biobank and Generation Scotland. *Translational psychiatry*. 9:14-14.

3. Townsend P, Phillimore P, Beattie A (1997): Health and Deprivation: Inequality and the North. *Revista Cubana De Higiene Y Epidemiología*. 35:48-50.

4. Chang CC, Chow CC, Tellier LC, Vattikuti S, Purcell SM, Lee JJ (2015): Second-generation PLINK: rising to the challenge of larger and richer datasets. *Gigascience*. 4:7-7.

5. Carroll RJ, Bastarache L, Denny JC (2014): R PheWAS: data analysis and plotting tools for phenome-wide association studies in the R environment. *Bioinformatics*. 30:2375-2376.

**Figure S1. A causal mediation analysis**


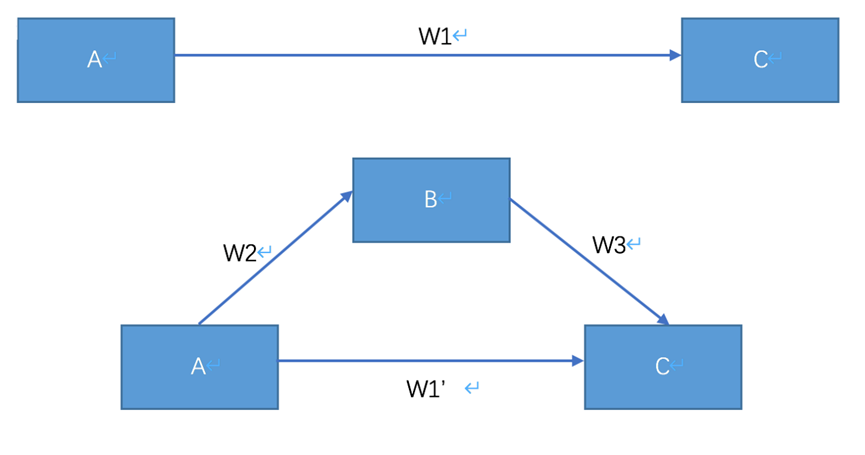


*A is the instrumental variable, C is the outcome variable, B is the mediate variable. W1 means the effect of A to C, W2 means the effect of A to B, W3 means the effect of B to C, W1’ means the effect of A to C with adjusted B.

**Figure S2. Genomic regions for depression and SUD.**


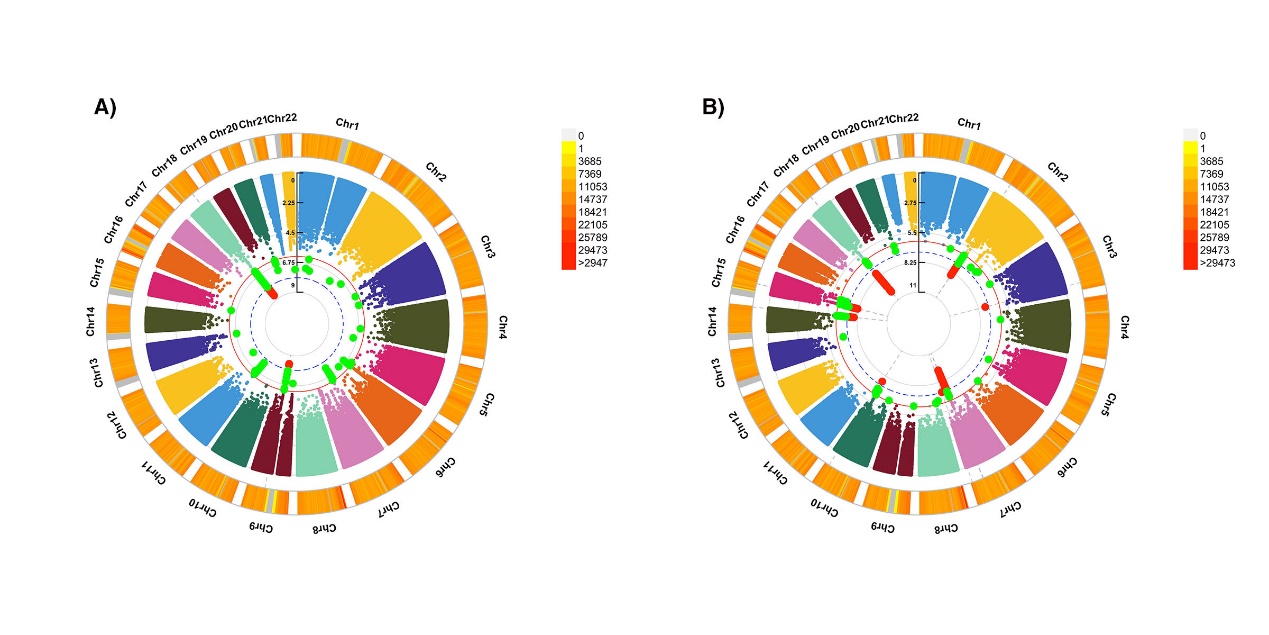
* From the center, the first circos depicts the –log_10_ P-values of the effect of the SNP allele to depression(A) or SUD(B). The second circos illustrates chromosome density. Red plots represent the *P* value <1.25 × 10^−8^ and green plots represent *P* value <5 × 10^−7^. The plots were generated using the “CMplot” R script (<https://github.com/YinLiLin/R-CMplot>). SUD, substance use disorders.

**Figure S3. Genomic regions for anxiety.**


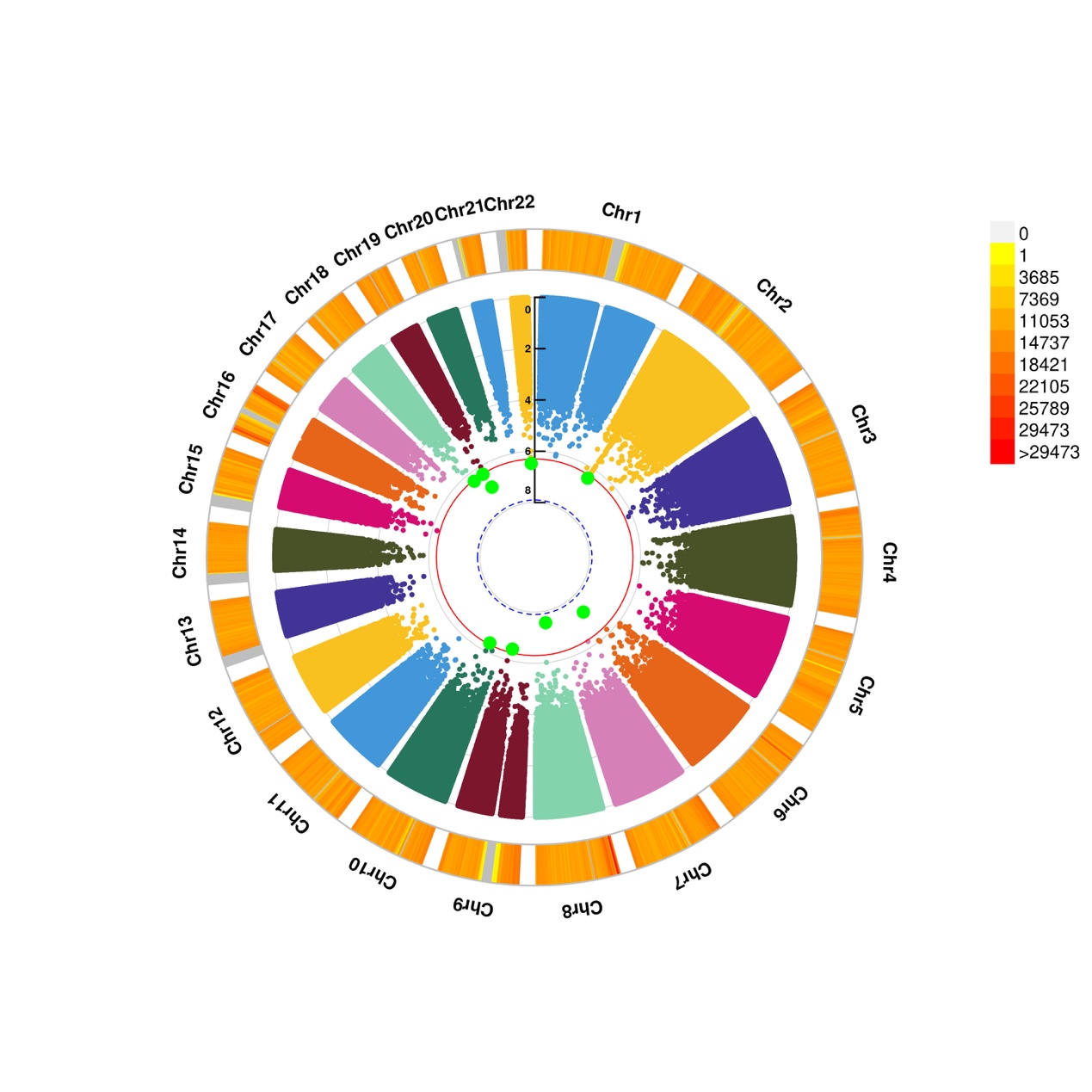


*From the center, the first circos depicts the –log_10_ P-values of the effect of the SNP allele to anxiety. The second circos illustrates chromosome density. Red plots represent the *P* value <1.25 × 10^−8^ and green plots represent *P* value <5 × 10^−7^. The plots were generated using the “CMplot” R script (<https://github.com/YinLiLin/R-CMplot>).

**Figure S4. Genomic regions for mental disorders.**


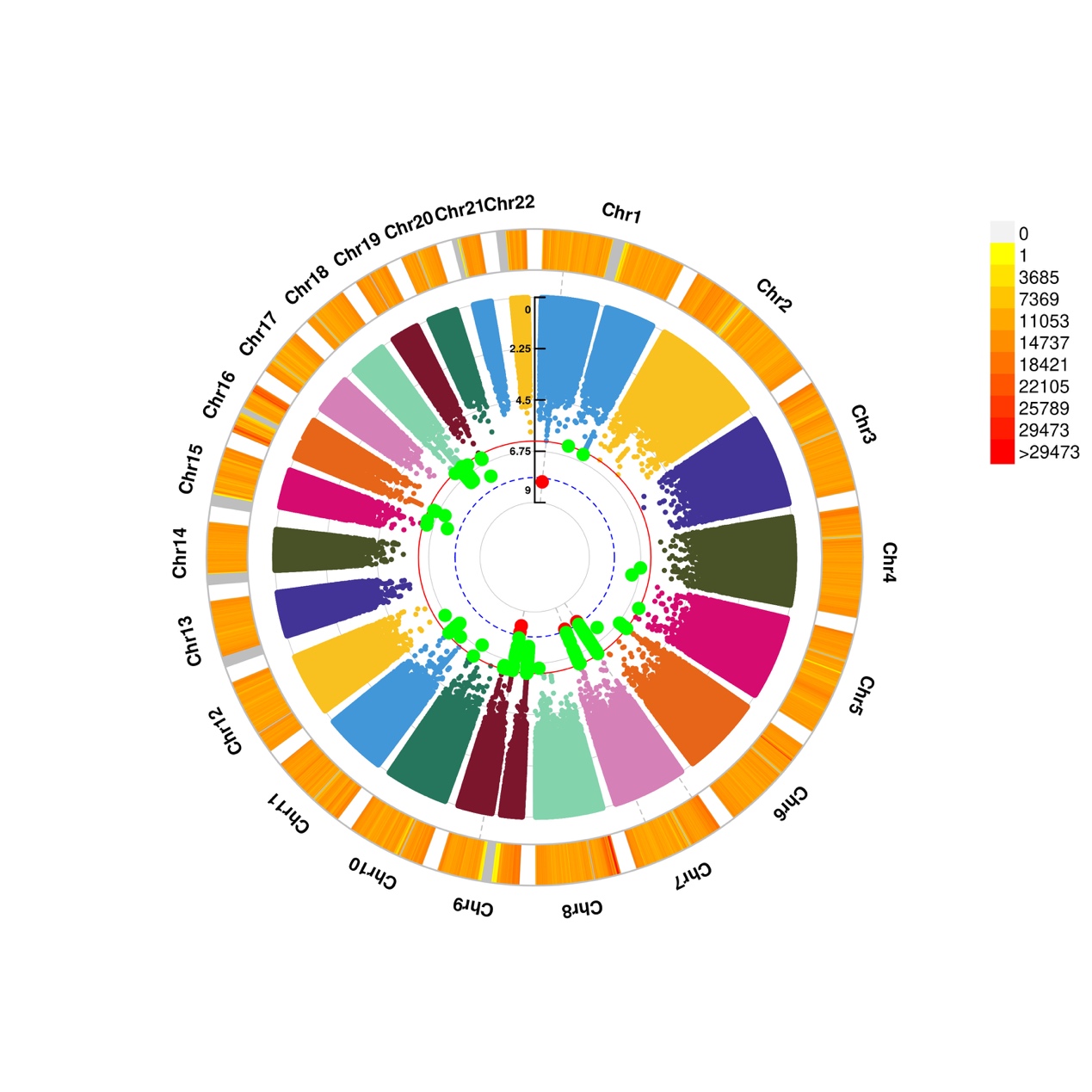


*From the center, the first circos depicts the –log_10_ P-values of the effect of the SNP allele to mental disorders. The second circos illustrates chromosome density. Red plots represent the *P* value <1.25 × 10^−8^ and green plots represent *P* value <5 × 10^−7^. The plots were generated using the “CMplot” R script (<https://github.com/YinLiLin/R-CMplot>).
